# Anatomical characteristics and visibility of mental foramen and accessory mental foramen: Panoramic radiography vs. cone beam CT

**DOI:** 10.4317/medoral.20585

**Published:** 2015-10-09

**Authors:** Juan Muinelo-Lorenzo, Juan-Antonio Suárez-Quintanilla, Ana Fernández-Alonso, Jesús Varela-Mallou, María-Mercedes Suárez-Cunqueiro

**Affiliations:** 1 PhD Student, Department of Stomatology, Medicine and Dentistry School, University of Santiago de Compostela, Spain; 2 Associate Professor, Department of Anatomy, Medicine and Dentistry School, University of Santiago de Compostela, Spain; 3 Professor and Chairman. Department of Social Psychology, Basic Psychology and Methodology, Psychology School, University of Santiago de Compostela, Spain; 4 Associate Professor, Department of Stomatology, Medicine and Dentistry School, University of Santiago de Compostela, Spain

## Abstract

**Background:**

The mental foramen (MF) is a small foramen located in the anterolateral region of the mandible through which the mental nerve and vessels emerge. The knowledge on the anatomic characteristics and variations of MF is very important in surgical procedures involving that area. The aim of this study was two-fold: firstly, to analyze the anatomic characteristics of MF and the presence of accessory mental foramen (AMF) using CBCT and, secondly, to compare the capability of CBCT and PAN in terms of MF and AMF visualization, as well as influencing factors.

**Material and Methods:**

A sample of 344 CBCT scans was analyzed for presence and characteristics (i.e. diameter, area, shape, exit angle) of MF and AMF. Subsequently, corresponding PANs were analyzed to ascertain whether MF and AMF were visible.

**Results:**

Out of the 344 patients, 344 (100%) MFs and 45 (13%) AMFs were observed on CBCT. Regarding gender, MF diameter and area, MF-MIB and MF-MSB distances, and exit angle were all significantly higher in males. Also, statistically significant differences were found in terms of age and dental status. Statistically significant differences in MF long and short diameters and MF area were found with respect to AMF presence (*p*=.021, *p*=.008, *p*=.021). Only 83.87% of the MFs and 45.83% of the AMFs identified on CBCT were also visible on PANs. MF diameter, shape, exit angle, and age had a significant influence on MF visualization on PAN (B=.43, *p*=.005; B=-.55, *p*=.020; B=.20, *p*=.008; B=.61, *p*=.005).

**Conclusions:**

PAN is not an adequate technique to properly identify MF and AMF. Diameter, shape, exit angle, and age are all factors influencing MF visualization on PAN images. For surgery involving the MF anatomical region, a preoperative radiological study with CBCT is of crucial importance to avoid complications.

**Key words:**Mental foramen, accessory mental foramen, mandibular anatomy, cone beam computed tomography, panoramic radiography.

## Introduction

The mental foramen (MF) is the opening through which the mental nerve exits the mandible and is usually located either between the roots of the first and second mandibular premolars or apical to the second premolar. The mental nerve represents one of the terminal branches of the mandibular nerve and divides into three branches supplying the lower lip, cheeks, chin, and the vestibular gingival of mandibular incisors (1). Although the anatomy of the mandibular nerve is well established, some anatomic variations have been reported which must be taken into consideration to avoid clinical complications. One of these variations is the accessory mental nerve, which passes through small foramina in the area surrounding the MF known as accessory mental foramina (AMF) (2).

The accessory mental nerve is a relevant anatomic structure in dental practice with particular importance for local anesthesia and surgical procedures involving this region, such as genioplasty, surgical rehabilitation after mandibular trauma, bone harvesting from the chin, root resection of mandibular premolars, and particularly the placement of dental implants (3,4). The incidence of permanent sensory disturbance to the lower lip after dental implant surgery in the MF area is reported to range from 7 to 10% (3). Sensation disturbance and severe pain may result if accessory mental nerve or mental nerve is entirely or partially damaged, and may lead to complications with significant impact on patients’ quality of life (1).

Two-dimensional radiographs are the most frequent imaging method in the dental field, but intraoral and rotational panoramic radiograph (PAN) images often fail to depict anatomic variations in the MF area (5). Using PAN, Al-Khateeb *et al*. (6) identified a considerable presence of AMF; however, Toh *et al*. (2) argued that AMF identification is difficult with intraoral and PAN radiographs because they are generally less than 1.0 mm in size. In contrast, presurgical tridimensional assessment using computed tomography (CT) could be a more useful tool for determining the presence of AMF. In particular, cone beam computed tomography (CBCT) provides sufficient resolution to allow accurate evaluation of osseous landmarks in the maxillofacial region and presurgical detection of AMF (4,7).

The aim of this study was two-fold: firstly, to analyze the anatomic characteristics of MF and the presence of AMF using CBCT and, secondly, to compare the capability of CBCT and PAN in terms of MF and AMF visualization.

## Material and Methods

The overall sample consisted of 357 consecutive patients who had both CBCT and PAN examinations, that were obtained from the Radiology Unit database at the Medicine and Dental School of the Santiago de Compostela University, Santiago de Compostela, Spain. The CBCT scans were performed from July 2008 to June 2014 for several clinical indications. Ethical approval for the study was obtained from the Galician Ethics Committee of Clinical Research (Ref. 2012/272). Written informed consent was obtained from all participants in the study.

The inclusion criteria consisted of the following: (1) PAN within a year of the CBCT; 2) the CBCT voxel size was 0.3 mm or less, and (3) whole mandibular body included in the CBCT volume. The exclusion criteria were as follows: (1) presence of any pathological and developmental conditions in the area of MF (i.e., tumors, cysts, fractures, or malformations); (2) presence of unerupted or partially erupted teeth in the anatomic region; and (3) presence of any artifacts or blurring affecting image quality.

-Image systems

CBCT examinations were performed using i-CAT® Model 17-19 (Imaging Science International, Hatfield, PA, USA), with a flat-panel detector of amorphous silicon, and an exposure protocol of 120 kVp, a current of 5 mA, 14.7 s, and the occlusal plane of patient was set parallel to the floor base by means of ear rods and a chin rest.

PAN images were performed using Orthophos® DS (Sirona Dental Systems GmbH, Bensheim, Germany) with a digital charge coupled device sensor. Exposure parameters were set at 80 kVp, 7 mA and 14.1 s and focus/sensor distance at 497 mm. The eye-ear plane of each patient was set parallel to the floor base. PAN exams were processed using a computed radiography system (Sidexis® neXt Generation; Sirona Dental Systems GmbH).

-Measurement procedure

Multi planar reconstructions from CBCT were jointly analyzed by two experienced researchers to identify MF and AMF. For this purpose, DICOM files were reconstructed on computer (Samsung R522, Samsung Electronics, South Korea) using 3D visualization and measurement software Carestream® CS 3D imaging software v3.2.12 (Carestream Health Inc., Rochester, NY, USA).

The location of MF was classified as being below or between the apices of inferior teeth from 1st molar to 1st premolar. The relation of the MF and AMF to the mandibular body was determined by the distances from the alveolar bone crest to the MF and AMF superior borders (MF-MSB, AMF-MSB), and from the MF and AMF inferior borders to the lower border of the mandible (MF-MIB, AMF-MIB). The crossing angle between the tangent to the vestibular mandibular surface and a parallel to the emerging direction of the mental nerve was considered the exit angle. The bone quality was determined using the Lekholm and Zarb classification, and Klemetti mandibular cortical index (C1: even and sharp; C2: semi lunar defects or endosteal cortical residues; and C3: heavy endosteal cortical residues and clearly porous).

Dental status was classified into three groups: dentate (6 or more remaining teeth), partially dentate (less than 6 remaining teeth) and edentulous.

**Figure 1:**
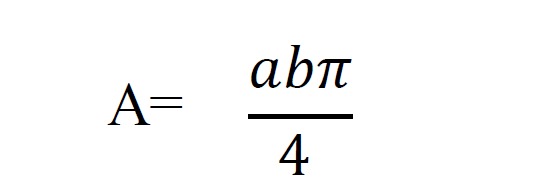
Formula oval area.

An AMF was defined as a buccal foramen showing continuity with the mandibular canal, excluding the mental foramen. AMF differed from MF in that it had a smaller diameter. Long and short axes of each MF and AMF were measured on sagittal images in CBCT exams; in addition, both MF and AMF areas were calculated using the formula: oval area, formula (Fig. 1). 

**Figure 2:**
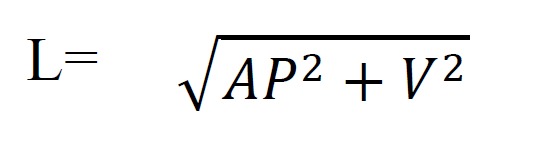
The formula the linear distance.

[a) and b) long and short diameters]. The position of AMF in relation to MF was assessed on CBCT axial and cross-sectional images. The AMF position in relation to MF was classified as follows: posterosuperior, posterior, posteroinferior, inferior, anteroinferior, anterior, and anterosuperior. The shortest distance between MF and AMF was measured in the horizontal (AP) and vertical directions (V) on sagittal images. The linear distance between MF and AMF was calculated using the formula (Fig. 2). 

In addition, the point of bifurcation from the mandibular canal and length of the accessory branch were also determined using axial images. CBCT measurements were carried out by an experienced researcher under constant conditions (dimly lit room and a 15.6-inch monitor). Intra-observer variability was assessed randomly on the CBCT images of 40 patients, which were repeated 1 month later.

Para-panoramic reconstructions from CBCT were obtained using Carestream® CS software, and rotational PANs were imported to image-processing and evaluation software (Photoshop 7.0; Adobe Systems, San Jose, CA). Two observers jointly assessed the appearance of MF on PAN images and classified MF into four categories as suggested by Yosue and Brooks (8): 1) continuous with the mandibular canal, 2) separated from the mandibular canal, 3) diffuse with indistinct borders, and 4) unidentified or non-visualized. Observers also assessed whether AMF was visible on PAN images. For this purpose, the location of AMF on CBCT para-panoramic reconstructions was used as a reference.

-Statistical analysis

Statistical analysis was performed using SPSS® v. 21.0 for Windows (IBM Corporation, Armonk, NY, USA). Descriptive statistics were compiled and expressed as mean and standard deviation. The level of intra-observer agreement was assessed for anatomical measurements using the intraclass correlation coefficient. The Pearson χ2 test and the t-test were used to test differences in frequency and differences in morphologic characteristics of MF and AMF between genders and age groups. The one-way ANOVA and post hoc Bonferroni multiple comparison tests were used to compare MF characteristics in relation to dental status. The effect of bone quality, and MF and AMF anatomical characteristics on MF and AMF visibility on PAN was assessed using binary logistic regression, adjusting for possible confounding variables. Differences were considered significant at *p*<.05.

## Results

The sample consisted of 344 CBCT scans selected out of the initial 357. From the total sample, three cases presented partially erupted or included teeth in the anatomic region, six volumes presented pathology (four patients had radiolucident images consistent with cysts or granulomas, one patient had lesions consistent with cherubism, and another had multiple dental inclusions and lesions suggesting cysts), and two CBCT scans and two PAN images lacked sufficient quality for analysis. The study sample consisted of 205 females (59.6%) and 139 males (40.4%) (mean age 47.44 ± 15.52; range: 13 to 86 years).

-Mental foramen and accessory mental foramen on cone beam computed tomography.

A total of 688 MFs presented a mean long diameter, short diameter and area of 4.44 ± 1.13 mm, 2.92 ± 0.75 mm, and 10.62 ± 5.00 mm2, respectively. The MF short diameter and MF area were significantly higher on the left side (*p*=.000, *p*=.013). The MF-MIB distance was 13.55 ± 1.06 mm, and MF-MSB distance was 11.42 ± 3.34 mm. The mean MF exit angle from the mandible was 53.45 ± 15.90°. Regarding gender, there were statistically significant differences in MF long diameter, MF short diameter, and MF area. In addition, both MF-MIB and MF-MSB distances, and the MF exit angle were statistically higher in males. Regarding age, MF-MSB distance was statistically higher in the younger age group. Conversely, the MF exit angle was statistically higher in the older age group ( Table 1). Regarding dental status, there were statistically significant differences in MF long diameter and MF area between dentate and edentulous patients (*p*=.013, *p*=.038). Also, MF-MSB distance was statistically higher in fully dentate patients (*p*=.000), while MF exit angle was statistically higher in edentulous patients (*p*=.000).

**Table 1:**
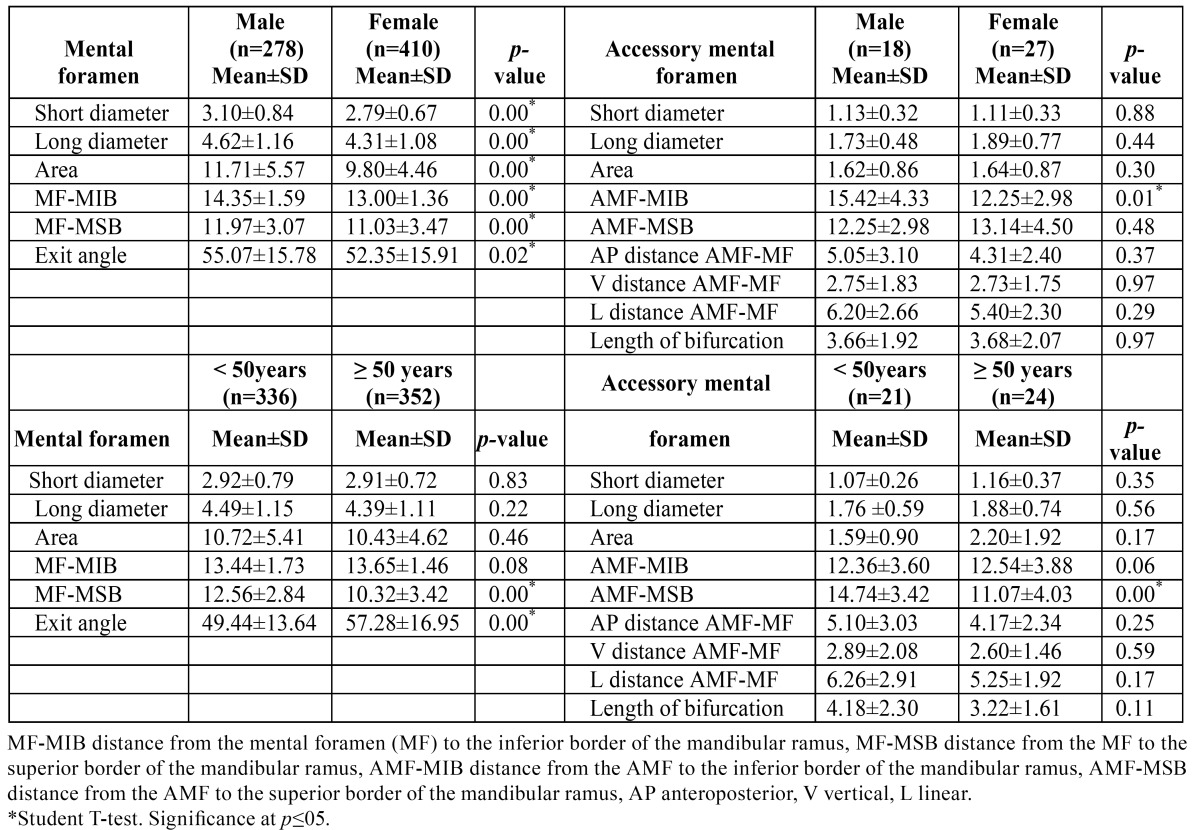
Morphological characteristics of mental foramen and accessory mental foramen by gender and age.

AMFs were observed in 45 (18 males and 27 females; 13.08%) of the 344 patients, and on 45 sides out of the total 688 (6.54%). Also, three cases had two AMFs on the same side. A total of 48 AMFs: 28 on the right side and 20 on the left side were observed. No statistically significant differences for the presence of AMF were found with respect to gender or side.

The mean long diameter, short diameter and area of AMF were 1.80 ± 0.66 mm, 1.12 ± 0.31 mm, and 1.58 ± 1.09 mm2, respectively The AMF-MIB distance was 11.72 ± 4.14 mm and the AMF-MSB distance was 13.52 ± 3.86 mm. No statistically significant differences in AMF characteristics were found between sides (*p*>.05). Regarding gender, no statistically significant differences were found in AMF long diameter, AMF short diameter or AMF area. The AMF-MIB distance was statistically higher in males. Regarding age, the AMF-MSB distance was statistically higher in the younger age group ( Table 1).

Regarding the relation between MF and AMF on CBCT, the position of AMF with respect to MF is shown in  Table 2. The mean AP, V and L AMF-MF distances were 4.46 ± 2.68 mm, 2.83 ± 1.82 mm, and 5.68 ± 2.41 mm, respectively. The length of bifurcation from mandibular canal was 3.62 ± 1.94 mm. No statistically significant differences were found in relation to gender, age group or side (*p*>.05).

**Table 2:**
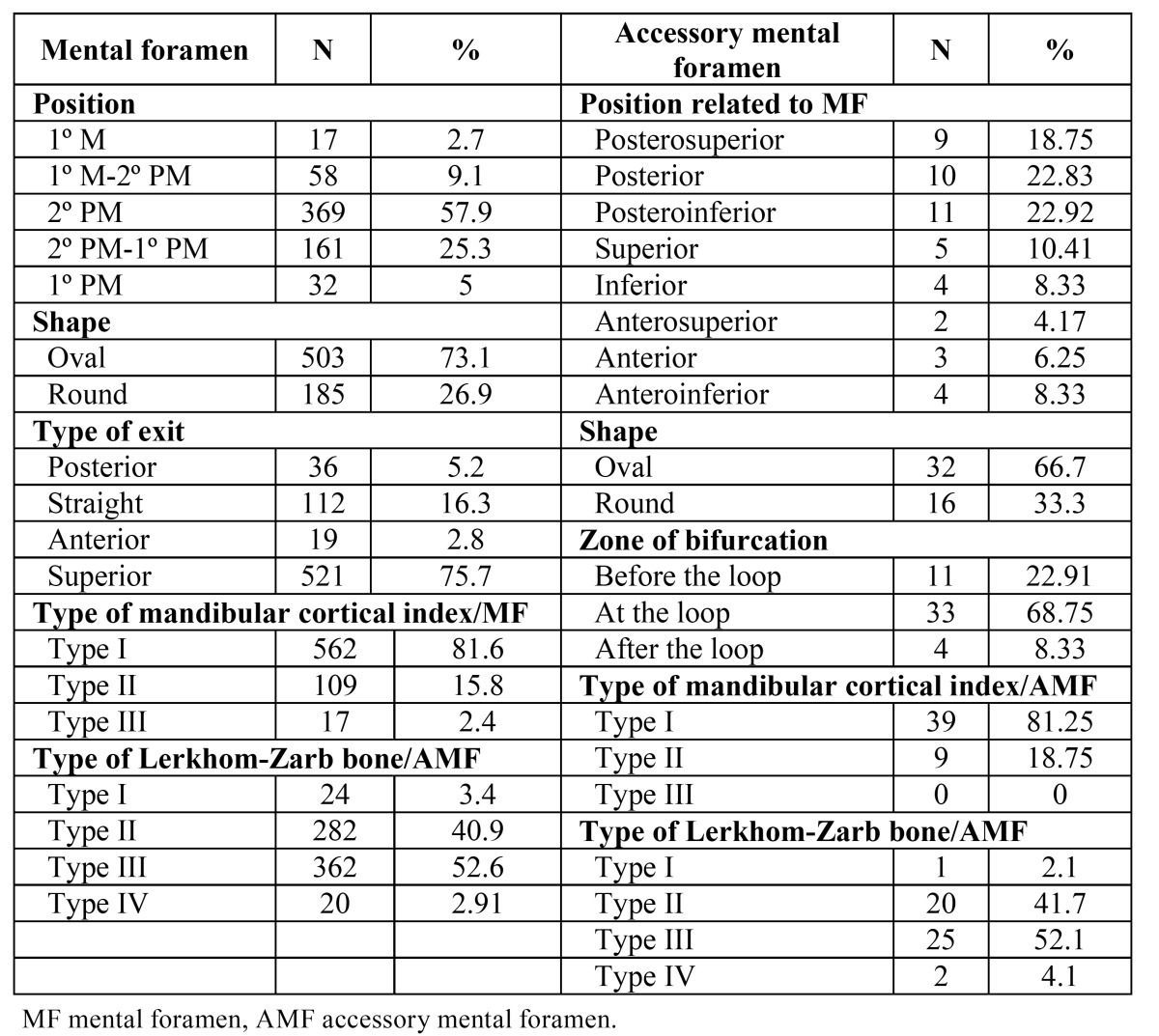
Morphological characteristics of mental foramen and accessory mental foramen.

The mean area of the 48 AMFs was 1.58 ± 1.09 mm2, and the rate of AMF area to MF area on the same side ranged from 3.92 to 77.81% (24.81 ± 18.96%). In addition, the rate of MF area on the side with the AMF to MF area on the side without AMF ranged from 16.41 to 265.43% (86.03 ± 52.33%).

The mean short diameter, mean long diameter and mean area of MF for the presence and absence of AMF were 2.66 ± 0.77 mm, 4.01 ± 1.20 mm, and 8.93 ± 5.20 mm2; and 2.93 ± 0.75 mm, 4.47 ± 1.12 mm and 10.71 ± 4.98 mm2, respectively. Statistically significant differences for the presence and absence of AMF were found (*p*=.021, *p*=.008, *p*=.021). Considering patients with AMF, the mean short diameter, mean long diameter and mean MF area on the side with AMF as compared to the side without AMF were 2.6 ± 0.7 mm, 4.0 ± 1.2 mm and 9.0 ± 5.2 mm2; and 3.0 ± 0.7 mm, 4.7 ± 0.9 mm, and 11.36 ± 4.5 mm2, respectively. Statistically significant differences for the presence and absence AMF were found (*p*=.014, *p*=.001, *p*=.013). The intra-observer agreement was assessed with intraclass correlation coefficient and presented values ranging from 0.72 to 0.97 (confidence interval of 95% ranging from 0.36 to 0.99).

-Mental foramen and accessory mental foramen on panoramic radiographs.

**Figure 3:**
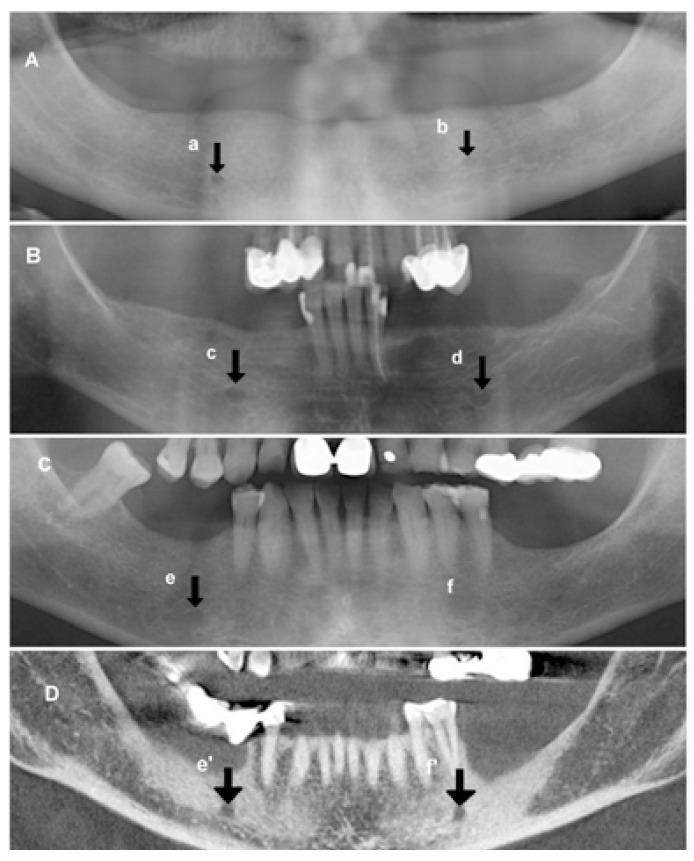
Mental foramen on panoramic radiographs (A,B,C): continuous (a), separated (c,d), diffuse (b,e), and unidentified (f) (Yosue and Brooks). Mental foramen on cone beam CT (D): The left mental foramen (f’) is visualized using para-panoramic reconstruction corresponding to patient C).

From the total MF sample, 577 out of 688 MFs were identified on PANs. This represents an MF visualization rate on PANs of 83.87% (Fig. 3). With respect to the visualized and non-visualized MF groups on PANs, a significant difference in short diameter, area, gender and age group was observed (*p*=.036, *p*=.041, *p*=.036, *p*=.033). No statistically significant differences were found in relation to dental status (*p*>.05).

The binary logistic regression was adjusted for age and gender. The morphological characteristics of MF, bone quality, age and gender were included in the model. MF short diameter, MF shape, MF exit angle, and age group were found to be statistically significant and did significantly influence MF visibility on PAN ( Table 3).

**Table 3:**
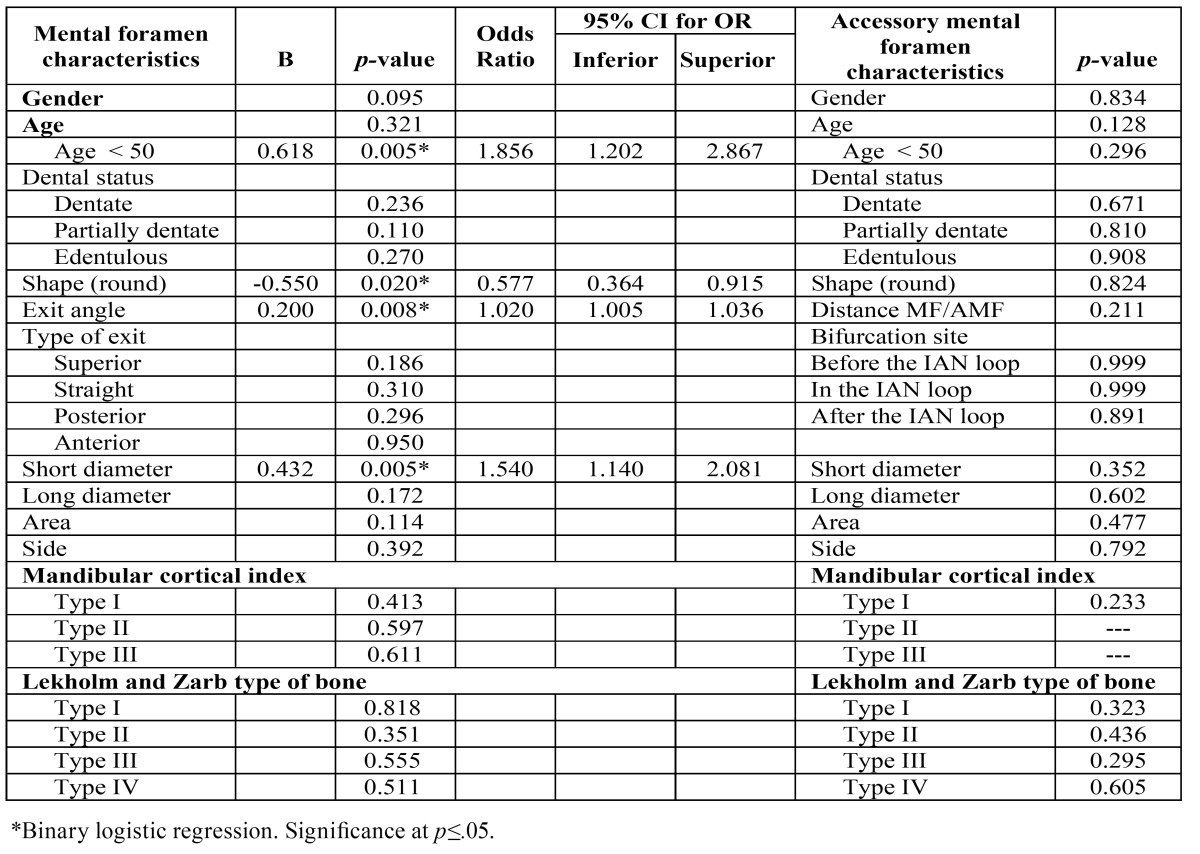
Effect of mental foramen, accessory mental foramen and bone characteristics on mental foramen and accessory mental foramen visibility using panoramic radiography.

**Figure 4:**
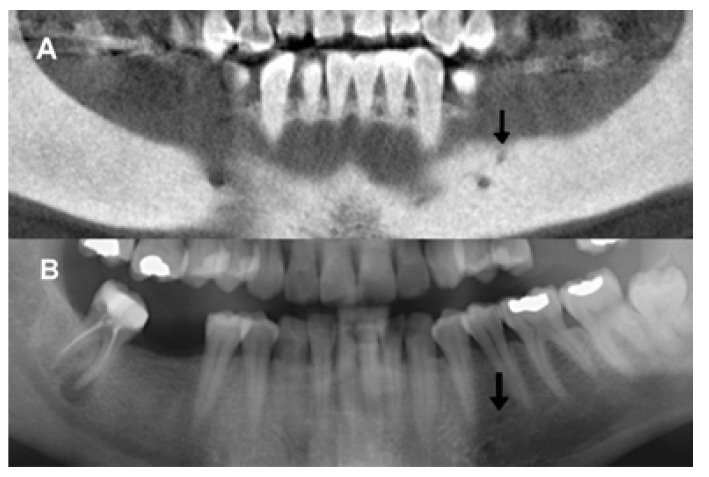
Accessory mental foramen using para-panoramic reconstruction from cone beam CT (A). Accessory mental foramen visualized on corresponding panoramic radiography (B).

**Figure 5:**
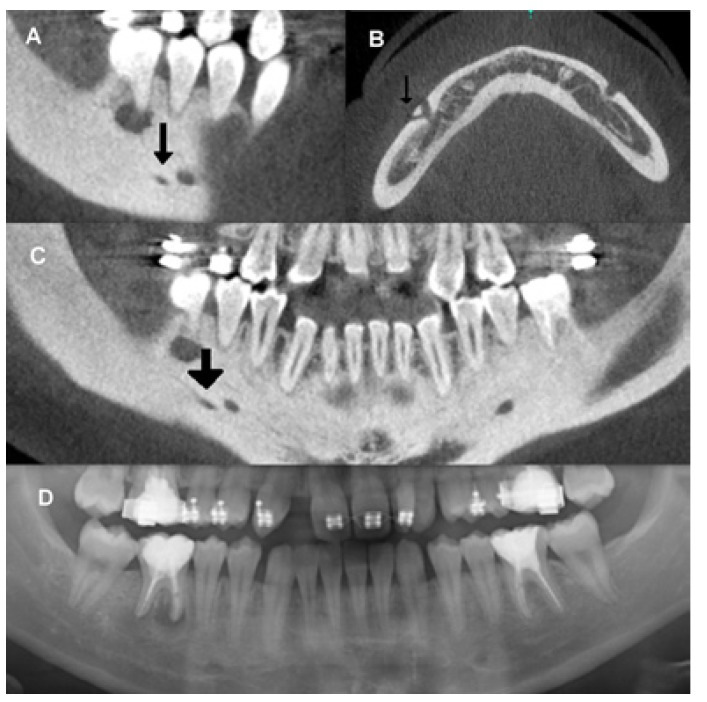
Comparison between cone beam CT images (A,B,C), and panoramic radiograph (A) of the same patient’s mandible. Sagittal (A), axial (B) and para-panoramic reconstructions revealed a posterior accessory branch of mental nerve emerging through an accessory mental foramen. Only the mental foramen is visualized with panoramic radiography (D).

On PAN images, the AMF was identified in 22 out of the 344 examinations (11 males and 11 females; 6.30% of the sample) and in 22 out of the 688 sides (3.15%). Out of the 48 AMFs observed on CBCT, 22 were identified using PAN (Fig. 4). This represents an AMF visualization rate on PAN images of 45.83% (Fig. 5). No statistically significant differences were found in relation to gender, age or dental status for AMF visibility on PANs. No significant differences in AMF and bone characteristics, gender or age groups were observed with respect to the visualized and non-visualized AMF groups on PAN images. The binary logistic regression included AMF morphological characteristics, bone quality, age and gender. None were found to significantly influence AMF visibility on PAN ( Table 3).

## Discussion

This study found an MF detection rate on PANs similar to previous research, but a slightly inferior level of clear visibility (8-10). Furthermore, our findings demonstrate that MF visibility on PAN is influenced by anatomic factors such as diameter, shape, exit angle, and age. Oval MF shape had the best visibility as did higher exit angle. The latter could be explained by the fact that the higher the angle the more perpendicular the incidence of x-ray beam. As stated in previous research, when the X-ray beam is projected parallel to the canal, visualization is more likely (11). With respect to age, younger patients presented better visibility. In this sense, Ngeow *et al*. (12) found 90.3% MF visibility in the 20-29 age group, while only 45% in the over 50 age group. A number of authors have suggested that these differences may be due to age-related bone changes (4,10,13,14,). However, bone characteristics were not found to influence visibility in the present study. Ngeow *et al*. (12) suggests that conventional radiographs may not be sufficient for presurgical assessment in older patients and may need to be supplemented with a 3D radiographic study.

In the scientific literature based on dry cranial studies, mean MF height ranges from 1.8 to 3.5 mm, mean MF width from 2.4 to 4.6 mm (15,16), and mean MF diameter from 2.1 to 5 mm (1,15,17). Research using CBCT has reported a mean MF height ranging from 3.0 to 3.7 mm, and a mean MF width from 3.2 to 3.4 mm (18-20). The present study has found MF dimensions in line with previous research, as well as discernible gender effects (19,20). Previous research involving children reported greater MF height and width in older patients (20). In contrast, we found no influence of age on MF dimensions in adults.

MF can present a variety of shapes (1). Several authors have reported oval shape to be the most common (17,21), representing 72% of MF (17). In line with these results, we bound oval to be the predominant shape, with rates similar to those reported by Gesherson *et al*. (17). However, other authors have found a predominance of round shape (6).

The MF-MIB and MF-MSB distances were in line with previous reports (15,16,19). Regarding gender, females presented shorter MF-MIB and MF-MSB distances, like most earlier studies (15,19). This is to be expected given gender differences in mandible size. With respect to age, MF-MSB distance was shorter in the older age group, perhaps from bone resorption due to more frequent dental absence. While Gershenson *et al*. (17) also reported differences, other authors found no influence of age on MF-MSB distance (19). With respect to dental condition, we found that dental absence caused a reduction in MF-MSB distance, like previous research (21,22).

The position of MF is located predominantly below the apex of second premolar or between the apices of the premolars (15,17,19). Our results show that MFs were mostly located below second premolar. We found no absence of MF, unlike earlier research with dry human mandibles (23).

The rate of occurrence of AMF was higher than other CBCT or CT studies (5,7,18,20,24-28), in line with Oliveira-Santos *et al*. (25). Previous cadaveric research showed the occurrence of AMF to be 5-30% (17,21,29). The rate of occurrence of AMF on PAN images was reported to be from 1.2 to 10% (6,28). However, both Imada *et al*. (5) and Katakami *et al*. (7) reported no AMF detection using PAN. The present study found a similar rate of AMF non-visibility on PAN as Naitoh *et al*. (4), between 45% and 50%. It may be that AMF detection on PAN images is more difficult than on CBCT para-panoramic images, because AMF images can overlap in various trabecular bone patterns (26). Naitoh *et al*. (30) found AMF detection to be difficult on PAN because it projects 3-D anatomic structures onto 2-D images, thus, superimposing both sides of mandible and cervical vertebrae.

Several studies claim that the position of AMF is usually inferior or posteroinferior to the MF (7,20,28), and that locating the MF position should be sufficient for most surgical procedures. However, the present study shows that up to 1/3 of AMF could be susceptible to injury during surgery because they are in a position above the MF, and this is supported by other authors (4,26).

Regarding the distance between the MF and AMF, a wide range from 0.67 to 15 mm (4,5,7,20,27) has been reported. In line with this, the present study showed a distance ranging from 2 to 13 mm. However, this distance did not play a role in AMF detection on PANs. Likewise, research regarding the visualization of accessory bony canal on PAN images reported no influence by the linear distance of AMF bony canal (26).

This study found that the presence of AMF influences the size of MF. On sides where AMF was present, MF was significantly smaller. Naitoh *et al*. also found MF size to be smaller on sides with AMF, but did not find statistical significance (4).

The mean AMF area in the present study was 1.5 mm2, which is similar to previous studies (4,26,30). We found AMF visualization on PANs not to be related to AMF size. Conversely, Naitoh *et al*. reported that AMF visualization on PANs was influenced by AMF size (26). In the present study, no cases of bilateral AMF were found. The literature reports that bilateral AMFs are scarce, ranging from 0.53 to 1.26% (4,5,7) However, we did find three cases with two AMFs on the same side. Apinhasmit *et al*. (15) reported this finding in 0.72% of cases.

The present study highlights the existence of certain factors influencing the MF visibility on PAN. Greater efforts are needed to determine factors influencing visibility of vital mandibular anatomical landmarks on PAN. Awareness of the advantages and limitations of different radiological techniques may be helpful in determining proper procedures before surgery.
